# Insulin eye drops for severe refractory chronic ocular graft-versus-host disease

**DOI:** 10.1038/s41409-024-02272-9

**Published:** 2024-04-10

**Authors:** V. Tahmaz, L. Menghesha, M. E. Stern, U. Holtick, C. Scheid, P. Steven

**Affiliations:** 1 Competence Center for Ocular GvHD, Department of Ophthalmology, University of Cologne, Faculty of Medicine and University Hospital Cologne, Cologne, Germany; 2 Department of Internal Medicine, University of Cologne, Faculty of Medicine and University Hospital Cologne, Cologne, Germany

**Keywords:** Graft-versus-host disease, Combination drug therapy

## To the Editor:

Ocular graft-versus-host disease (oGVHD) is a common and severe complication after allogeneic stem cell transplantation (aSCT) and affects up to 60% of patients who undergo this procedure [1]. Besides acute forms, oGVHD mainly presents as chronic disease affecting the entire ocular surface and adnexae. Symptoms include ocular irritation and discomfort, photophobia, pain, and decreased visual acuity, clinical signs include inflammation of the eyelids, conjunctiva, and cornea with epitheliopathy that may progress to corneal neovascularization and opacification. In severe cases, corneal ulcerations, perforation, and loss of the eye may occur [2].

Therapy of oGVHD is adjusted to disease severity, using preservative-free artificial tears and topical corticosteroids often combined with topical ciclosporine. Supplemental options like contact lenses, punctual plugs, and lid margin therapy exist. With these treatments moderate cases can be controlled and ocular surface disease improved. Topical anti-inflammatory treatment is often continued for years and sometimes combined with systemic immunosuppression, especially when other organs are affected by GvHD. There is a subset of patients with therapy-refractive oGVHD and persisting inflammatory activity. In these cases, regenerative agents such as serum eye drops or amniotic membrane transplantation may provide additional effects by promoting wound healing and improving barrier function. Still some cases remain therapy refractive, present persisting inflammatory activity and experience severely impaired quality-of-life due to loss of vision and pain. These patients are at increased risk for complications like corneal melting and perforation. For these patients there is an unmet need for additional treatment options.

Insulin eye drops (INED) have been reported in 1945 by Aynsley as a treatment for corneal ulcers due to diabetic neurotrophic keratitis, demonstrating potential for promoting epithelial regeneration and improving corneal barrier function [3]. Multiple studies have shown a benefit of INED in the treatment of severe dry eye disease or persistent epithelial defects with no relevant side effects [4–6]. The use of topical insulin could be a promising option for therapy-refractive oGVHD and was suggested as last-resort individual healing attempts (IHA) to individual patients in our clinic.

A retrospective analysis of all patients receiving INED for severe chronic ocular GVHD in our institution was performed. Seven patients with bilateral severe chronic oGvHD (NIH Grade III) after aSCT were treated with INED at 1 IU/ml after informed consent to “IHA”, meaning an off-label drug application after failure of all approved treatment options. One cycle included 6 weeks of INED four times daily. Before this therapy, all patients had been treated for chronic ocular GVHD according to GVHD guidelines of the German Society of Ophthalmology, including artificial tears, topical corticosteroids, topical cyclosporine, and serum eye drops. Two patients continued use of cyclosporine eye drops, six patients continued use of dexamethasone eye drops and all patients continued use of serum eye drops during insulin application.

Regarding systemic manifestations of GVHD, one patient suffered from hepatic GVHD, one was diagnosed with chronic GVHD liver, skin, and oral mucosa, one patient presented with chronic GVHD in skin, mucosa, and musculoskeletal apparatus.

Data collected from patient files included best corrected visual acuity, fluorescein staining of cornea and conjunctiva, ocular surface disease index (OSDI) questionnaire, and photographic documentation as well as concomitant therapy. Data were collected from two consecutive examinations before (pre 2 and pre 1) and two examinations after insulin application (follow-up 1 and follow-up 2). First-time application of insulin was carried out in our outpatient clinic together with measurement of blood glucose concentration 1 h after first instillation. During therapy, patients were examined weekly first and bi-weekly later, if appropriate. Four patients did not receive any systemic immunosuppressive therapy, one received 100 mg cyclosporine daily, one received ruxolitinib 5 mg daily, one received ruxolitinib 10 mg daily – all systemic therapy regimes had remained unchained for at least 1 year before topical insulin therapy. No patient had a history of diabetes. Statistical analysis was performed using Mann–Whitney *U* test in Python (*p* values for baseline versus follow-up 1 at 0.001, for baseline versus follow-up 2 at 0.0002).

**Fig. 1:**
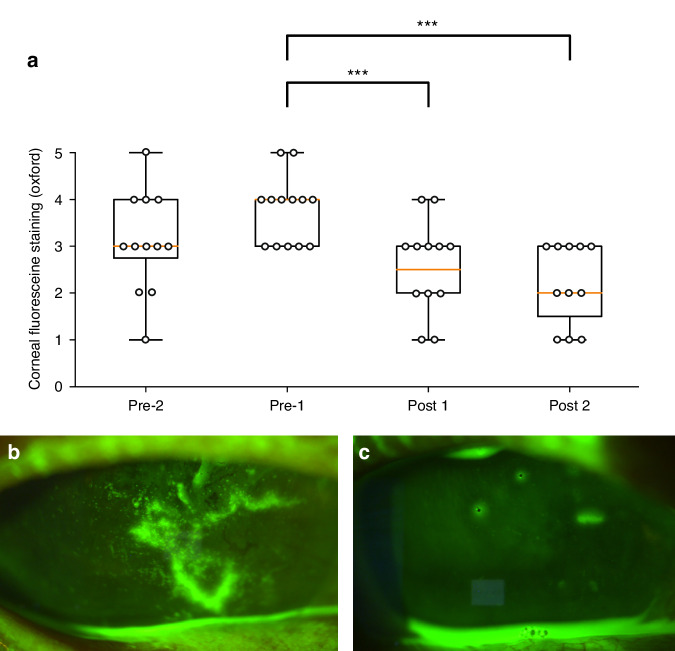
Graphical presentation of corneal fluorescein staining grade before and during therapy with insulin eye drops and two exemplary slit lamp photographs of the same eye before and during therapy with insulin eye drops. **a** Box plot of corneal fluorescein staining grade at two visits before (pre 2 and pre 1) and two consecutive visits after insulin eye drop application (post 1 and 2). Individual data points have been marked as dots. Asterisks mark statistical significance with *p* < 0.05. Sixty-nine years old male patient 5 years after aSCT for chronic lymphoblastic leukemia, slit lamp image before (pre 1, **b**) and after (post 1, **c**) 6 weeks of insulin eye drop application.

Seven patients suffering from bilateral severe therapy-refractory chronic oGVHD, NIH Grade III were treated with INED. Median time from aSCT was 9.5 years (2–16 years). aSCT had been performed for Acute Myeloid Leukemia (*n* = 2), Acute Lymphoblastic Leukemia (*n* = 2), Chronic Myeloid Leukemia (*n* = 2), and Chronic Lymphoblastic Leukemia (*n* = 1). The median age was 52 years (35–69), female to male ratio was 1:6. All patients had received topical corticosteroids, artificial tears, and serum eye drops up to 10×/day without improvement. Two patients had applied 0.1% cationic topical cyclosporine. All patients reported good tolerance of INED without adverse reactions, blood glucose measurements were within the normal range. At both pre-IHA visits (pre 2 and pre 1) visual acuity was 0.44 and 0.47, OSDI scores were both 70.83 and 70.58 and corneal fluorescein staining were 3.08 and 3.79, respectively. After a mean 5.4 weeks of insulin eye drop therapy (range 3–7), visual acuity was 0.29 and OSDI scores were 64.73 with no significant change, NIH grading remained III. Corneal fluorescein staining improved significantly (Fig. 1, follow-up 1) to 2.5. After the next follow-up examination and discontinuation of INED (mean 10.2 weeks, 5–20), visual acuity and OSDI did not show statistical differences, corneal staining remained significantly improved at Oxford grade 2.18 (Fig. 1, follow-up 2).

Existing therapeutic options usually suffice in controlling chronic oGvHD and surgical options allow management of acute complications, but there is an unmet need for additional medical therapy in severe therapy-refractive cases.

Our first experiences with INED as a treatment in these cases showed promising results and improved epitheliopathy in all patients. In further follow-up examinations after cessation of therapy, no adverse effects were registered over 6 months.

Although all patients reported subjective improvement of ocular discomfort during therapy, there were no significant changes in OSDI scores. However, corneal staining demonstrated statistically significant improvement over 6 weeks, showing a wound healing effect. Improvement lasted for weeks after discontinuation of insulin therapy.

The mode-of-action of INED on corneal epithelium in oGVHD has not been investigated. Insulin is present in tear fluid [7], regulates cellular metabolism and growth of corneal epithelial cells through receptors such as insulin receptor (ISNR) or insulin-like growth factor receptor (IGF-1R) [8]. It is not involved in corneal glucose uptake which is facilitated by glucose transporter-1 (GLUT1) [9]. Experimental studies, using mouse models of oGVHD [10] are under way to investigate insulin levels in tear film and its cellular effects in different phases and severities of oGVHD and to study the mode-of-action that we see in the first clinical applications.

Overall, the compelling efficacy of INED in the treatment of patients with severe refractory chronic oGvHD implies to consider this as an additional option for topical treatment.
